# Epigenetic regulation of key gene of PCK1 by enhancer and super-enhancer in the pathogenesis of fatty liver hemorrhagic syndrome

**DOI:** 10.5713/ab.23.0423

**Published:** 2024-04-23

**Authors:** Yi Wang, Shuwen Chen, Min Xue, Jinhu Ma, Xinrui Yi, Xinyu Li, Xuejin Lu, Meizi Zhu, Jin Peng, Yunshu Tang, Yaling Zhu

**Affiliations:** 1 Department of Pathophysiology, Anhui Medical University, Hefei, 230032, China; 2 Laboratory Animal Research Center, College of Basic Medical Science, Anhui Medical University, Hefei, 230032, China

**Keywords:** ChIP-Seq, Fatty Liver Hemorrhagic Syndrome, H3K27ac, RNA-Seq, Super-enhancer

## Abstract

**Objective:**

Rare study of the non-coding and regulatory regions of the genome limits our ability to decode the mechanisms of fatty liver hemorrhage syndrome (FLHS) in chickens.

**Methods:**

Herein, we constructed the high-fat diet-induced FLHS chicken model to investigate the genome-wide active enhancers and transcriptome by H3K27ac target chromatin immunoprecipitation sequencing (ChIP-seq) and RNA sequencing (RNA-Seq) profiles of normal and FLHS liver tissues. Concurrently, an integrative analysis combining ChIP-seq with RNA-Seq and a comparative analysis with chicken FLHS, rat non-alcoholic fatty liver disease (NAFLD) and human NAFLD at the transcriptome level revealed the enhancer and super enhancer target genes and conservative genes involved in metabolic processes.

**Results:**

In total, 56 and 199 peak-genes were identified in upregulated peak-genes positively regulated by H3K27ac (Cor (peak-gene correlation) ≥0.5 and log2(FoldChange) ≥1) (PP) and downregulated peak-genes positively regulated by H3K27ac (Cor (peak-gene correlation) ≥0.5 and log2(FoldChange)≤−1) (PN), respectively; then we screened key regulatory targets mainly distributing in lipid metabolism (*PCK1*, *APOA4*, *APOA1*, *INHBE*) and apoptosis (*KIT*, *NTRK2*) together with MAPK and PPAR signaling pathway in FLHS. Intriguingly, *PCK1* was also significantly covered in up-regulated super-enhancers (SEs), which further implied the vital role of *PCK1* during the development of FLHS.

**Conclusion:**

Together, our studies have identified potential therapeutic biomarkers of PCK1 and elucidated novel insights into the pathogenesis of FLHS, especially for the epigenetic perspective.

## INTRODUCTION

Fatty liver hemorrhagic syndrome (FLHS) is one of the highest non-communicable diseases causing mortality in laying hens worldwide, occurring with lipid deposition and liver hemorrhage [1,2]. Research has shown that overfeeding of laying hens results in nutritional overload which is considered a key factor in FLHS development, and a model of FLHS induced by high-fat diet (HFD) has been successfully established [3]. Moreover, the usual pathological characteristics and clinical symptoms in FLHS of laying hens include inflammation and oxidative stress reaction in the liver [4]. Yao et al [5] revealed that activation of AMPKα signaling could alleviate oxidative stress and inflammatory responses induced by a high nutrient diet in laying hens. Although studies have provided methods to reduce the occurrence of FLHS, the exact mechanism of how FLHS occurs in laying hens, especially epigenetic mechanisms, remains obscure.

Recently, studies have revealed that epigenetically induced changes in gene function are increasingly important in the progression of the disease, among which histone modifications affecting the transcriptional expression of target genes by regulating genomic elements are a key part of epigenetic inheritance [6]. Histone H3 lysine 27 acetylation (H3K27ac), a representative epigenetic marker, has been reported to identified active enhancer and super enhancer in activation of gene expression [7]. Some research found that enrichment of H3K27ac may be associated with altered cholesterol metabolism [8]. A few studies further revealed that the epigenetic marker H3K27ac was mainly expressed at lipid-rich loci, associated with epigenetic remodeling, the activation of the target genes and transcription factors, which participated in pathways connected with the regulation of adipogenesis and lipid metabolism [9]. Additionally, an imbalance of H3K27ac modification contributed to the development of various liver diseases such as non-alcoholic fatty liver disease (NAFLD) [10]. Super-enhancers (SEs) are composed of multiple neighboring enhancers that synergistically regulate gene transcription as well as drive specific biological functions of diseases, while H3K27ac widely serves as the characteristic of the SEs [11]. However, the active enhancers and SEs marked by H3K27ac in FLHS have been rarely explored.

In this study, we adopted H3K27ac target chromatin immunoprecipitation sequencing (ChIP-seq) and RNA sequencing (RNA-Seq) to identify differentially acetylated peaks and differentially expressed genes (DEGs) in the liver, as well as to probe SEs, which further revealed relevant candidate genes and provided potential pathogenesis and therapeutic targets.

## MATERIALS AND METHODS

### Ethics and consent

The Ethics Committee of Anhui Medical University approved all the experimental procedures with the Chinese Ministry of Agriculture’s defined standards for the care and use of experimental animals (LLSC20200014).

### Laboratory animals and tissue collection

In this study, there are 60 healthy Hy-Line Brown laying hens (body weight: 1.1 to 1.8 kg, age: 145 days). After one week of adaptive feeding, the experimental animals were randomly divided into control and FLHS groups, with 30 laying hens in each group. The FLHS group was fed with HFD, and a normal chow diet was fed to the control group [3]. After fifty days of feeding, we randomly selected 3 chickens from each of the two groups. The liver tissues and plasma samples were dissected for further research. Fresh livers were stored at −80°C for backup.

### Histopathological examination

We used 10% formalin to fix, and paraffin embedded the portion of the collected liver tissues. The embedded tissue was stained with oil red O (0.5% in isopropanol diluted 6:4 in water) for 18 min. The Zeiss Axioskop 40 microscope was pictured for microscopy images.

### RNA-Seq and analysis of differential expressed genes

We used TRIzol reagent (Invitrogen, Carlsbad, CA, USA) to extract total RNA from the liver tissues of three FLHS individuals and three normal individuals. Library preparation was performed using the NEBNex Ultra Directional RNA Library Prep Kit for Illumina (NEB, Ipswich, MA, USA) and RNA-Seq was performed using the Illumina HiSeq 4000 platform with 150 bp paired-end sequencing. Then STAR-2.5.3a was used to map the filtered reads in the chicken reference genome Gallus_gallus-6.0 (Ensembl) [12] and mapped reads were counted using feature Counts software v2.0.6 [13]. Gene with low expression across samples (when 80% of the samples have counts below 2) was excluded before DESeq2.

The fragments per kilobase of transcript sequence per million base pairs (FPKM) normalized read counts; the DESeq2 R package (version 1.30.1) was applied to analyze differential gene expression [14] and Benjamini and Hochberg’s approach for controlling false discovery rates was used to adjust the results of p-value. Threshold of DEGs: |log2(FoldChange)|≥1 and p-value≤0.05.

### ChIP-Seq and analysis of differentially H3K27-acetylated peaks

As for the chromatin immunoprecipitation (ChIP), we use the samples as same as the samples applied in RNA-seq. The experimental procedure according to the SimpleChIP Enzymatic Chromatin IP Kit (Magnetic Beads, 9005). We used the Burrows–Wheeler Aligner (BWA) (version 0.7.17-r1188) [15] to map clean reads to the chicken reference genome Gallus_gallus-6.0 (Ensembl). Then we identified H3K27ac enriched regions using MACS (version 2.1.0) by setting the q-value threshold at 1e-5 [16]. Then bedtools (version 2.27.0) was used to merge all peaks files into one group file [17]. To quantify acetylated peaks between normal and FLHS chickens, we sorted and analyzed all BAM files using the ‘bedcov’ utility from samtools (version 1.2) [18]. Finally, we obtained differentially H3K27-acetylated regions with DESeq2 R package (version 1.30.1) and considered |log2(FoldChange)|≥1 and p-value≤0.05 regions as differential peaks as we previously published article [19]. ChIP-Seq heatmaps and profiles were generated by deeptools (version 3.5.1) [20]. Super-enhancers were performed following the methods reported by Whyte et al [21].

### Genome-wide “four-way” of altered H3K27 acetylation and differential gene expression

To conduct an integrative analysis of RNA-seq and ChIP-seq, we calculated the correlation between DEGs and differential peaks [19]. Next, we defined the correlated peak-gene with Pearson correlation coefficient higher than 0.5. Then we adopted genome-wide “four-way” to identify whether the differential peaks could regulate target genes in a positive or negative way by using the threshold of |Cor (peak-gene correlation)| ≥0.5 and |log2(FoldChange)|≥1. Among that PP was represented as upregulated peak-genes positively regulated by H3K27ac (Cor (peak-gene correlation) ≥0.5 and log2(FoldChange) ≥1); PN was known as downregulated peak-genes positively regulated by H3K27ac (Cor (peak-gene correlation) ≥0.5 and log2(FoldChange) ≤−1), NP as the upregulated peak-genes negatively regulated by H3K27ac (Cor (peak-gene correlation) ≤−0.5 and log2(FoldChange) ≥1); NN as the downregulated peak-genes negatively regulated by H3K27ac (Cor (peak-gene correlation) ≤−0.5 and log2(FoldChange) ≤−1).

### Gene ontology and pathway enrichment analyses

DAVID (https://david-d.ncifcrf.gov/) was performed to identify overrepresented gene ontology (GO) and pathways of hyper- and hypo-acetylated peaks (groups of PP and PN, respectively), and the overlapped DEGs of human, rat and chicken RNA-seq data in multiple comparative analysis of fatty liver disease, as well as up-regulated SEs target genes. The p-value ≤0.05 was regarded as enriched in Go terms and pathways.

### ChIP-qPCR and RT-PCR validation

ChIP-qPCR assays were performed using the SimpleChIP Plus Enzymatic Chromatin IP Kit (Magnetic Beads, 9005; Cell Signaling Technology (CST), Danvers, MA, USA) with 500 μg chromatin from the same samples used in RNA-seq and 5 μg anti-H3K27ac antibody. ChIP-qPCR by calculating the fold enrichment of the positive locus sequence in ChIP DNA over the negative locus: fold enrichment = 2^−ΔΔCt^ [19]. Then in order to get the mRNA expression of potential target genes, we used real-time polymerase chain reaction (PCR) as reference to published articles in our group [19].

### Statistical analysis

GraphPad Prism 9 (GraphPad Software, San Diego, CA, USA) and R-4.2.2 (https://cran.r-project.org/) were used for statistical analysis. The correlation coefficients of peak-genes and samples were determined using Pearson correlation analysis and Spearman correlation analysis, respectively. The t-test was applied. Error bars in the Figures represented standard deviation.

## RESULTS

### Pathological anatomy and histomorphological differences of liver tissues from FLHS and normal chickens

**Figure 1:**
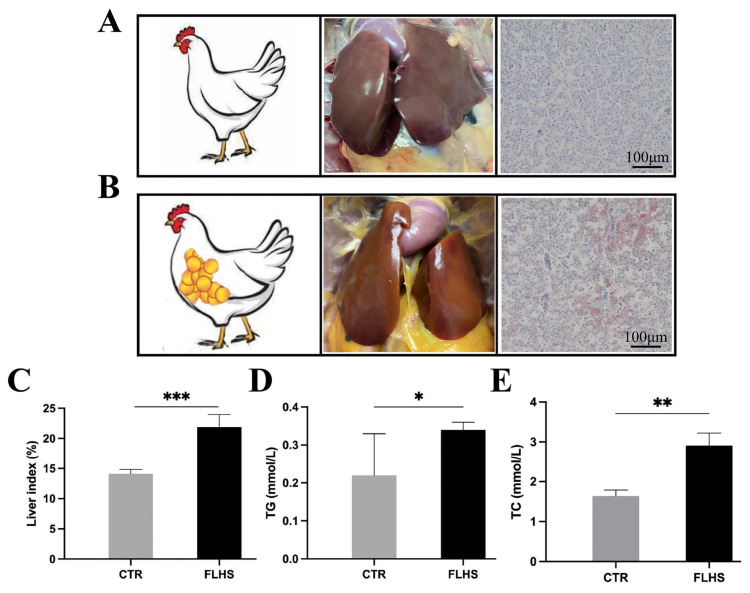
Liver histopathological images and related lipid metabolism parameters in the normal and FLHS chickens. (A) Anatomy and histopathology of the liver of normal laying hens. (B) Anatomy and histopathology of the liver of laying hens with FLHS. (C–E) Effect of FLHS on live index, TG and TC in chickens. FLHS, fatty liver hemorrhage syndrome; TG, triglycerides; TC, total cholesterol. Liver index (‰) = Humid weight of liver/body weight. * p≤0.05, ** p≤0.01, *** p≤0.001.

To investigate the pathogenesis of FLHS, we constructed HFD-induced FLHS model as the dietary background based on published articles [3]. As shown in Figure 1A–1B, the livers of FLHS group were yellowish brown, large and brittle accompanied by thick fat pads in the lower abdomen of the liver compared to normal groups. Moreover, histopathological sections of the FLHS group showed more obvious fat droplets attaching to the cytoplasm of hepatocytes (Figure 1A–1B). In addition, liver index (p≤0.001), liver triglycerides (TG) (p≤ 0.05) and total cholesterol (p≤0.01) were significantly higher in the FLHS group than that of normal group (Figure 1C–1E), further indicating the successful construction of FLHS model in this study.

### ChIP-Seq and RNA-Seq sequencing process and correlations of samples

**Figure 2:**
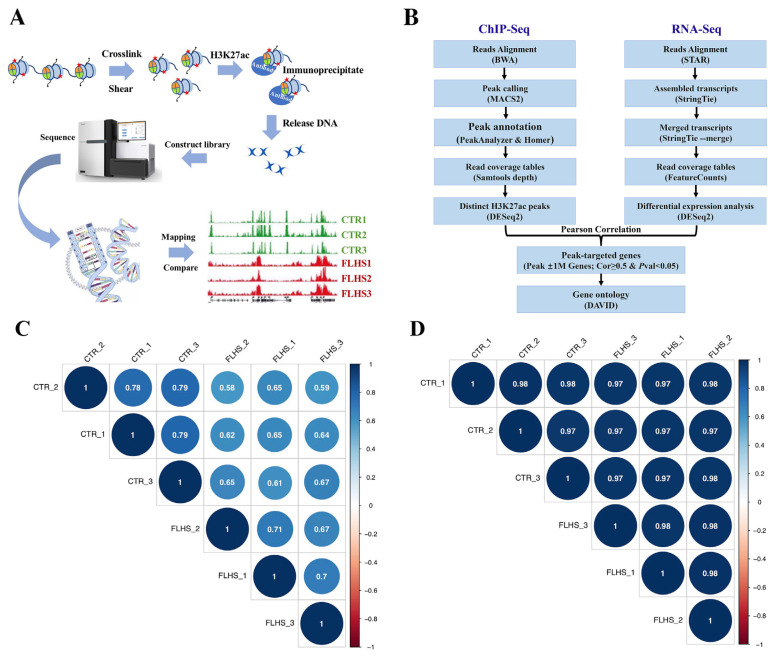
ChIP-Seq and RNA-Seq Sequencing samples. (A) The basic rationale of ChIP-Seq. (B) Integrative analysis workflow of RNA-Seq and ChIP-Seq. (C) Spearman correlation analysis of biological duplicate samples in ChIP-Seq. (D) Spearman correlation analysis of biological duplicate samples in RNA-Seq.

To investigate the impact of epigenetic and transcriptional factors on FLHS, we collected liver tissue samples for RNA-Seq and ChIP-Seq analyses. Figure 2A illustrates the underlying principle of ChIP-Seq, while Figure 2B demonstrates the integrative bioinformatics analysis of RNA-Seq and ChIP-Seq. This analysis facilitated the identification of the H3K27ac peaks and differential gene expression, enabling the identification of target genes associated with these peaks. Additionally, functional enrichment analysis was performed in parallel to further investigate the biological significance of these findings.

After that, we examined the quality of the data to ensure the reliability of our data. The average number of reads uniquely mapped to the reference genome of the input and H3K27ac samples in ChIP-Seq was 32.7 M (29.0 to 36.4 M) and 32.5 M (29.7 to 39.1 M), respectively. And in RNA-Seq, the number was 47.5 M (45.7 to 51.1 M) (Supplementary Table S1). In addition, Spearman analysis showed intra- and inter-group correlation coefficients were greater than 0.6 for both RNA-Seq and ChIP-Seq, indicating that the sample data were of high quality and could be used for further analysis (Figure 2C–2D).

### Genome-wide profiling of H3K27ac associated with differentially expressed genes

**Figure 3:**
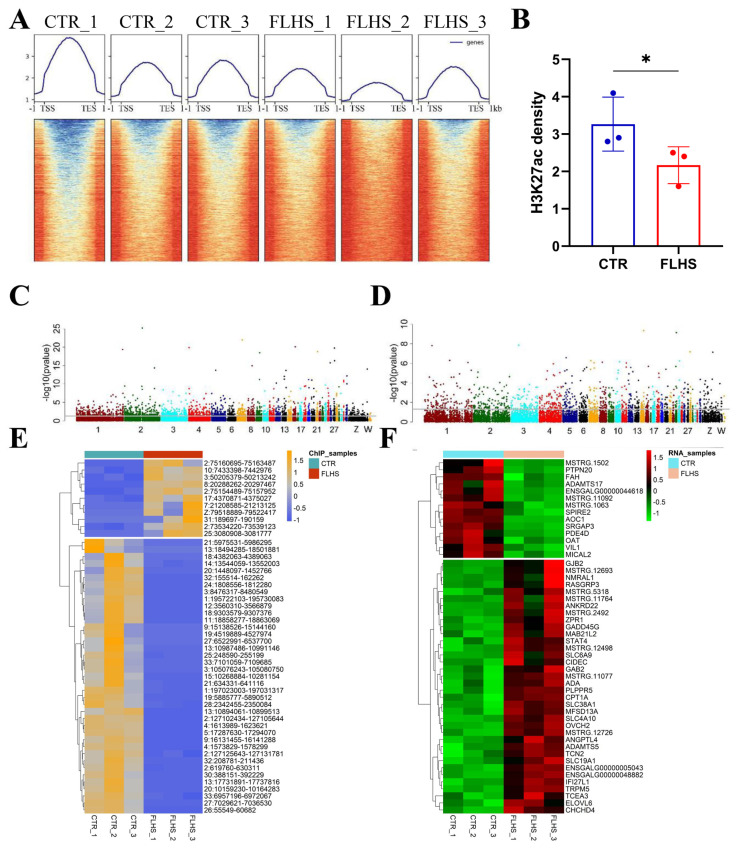
Profiles of differential H3K27ac peaks and differentially expressed genes associated with FLHS in the liver. (A) Profiles and heatmaps showing the H3K27ac signal at 1 kb near transcription start site (TSS) and transcription termination site (TES) in the liver between normal and FLHS chickens (n = 3/group). (B) Quantification of H3K27ac density in each group (n = 3/group). (C) Manhattan plot showed the distribution of H3K27ac peaks on different chromosomes (grey line, p<0.05). (D) Manhattan plot showed the distribution of genes on different chromosomes (grey line, p<0.05). (E–F) Heatmaps of top 50 significant differential H3K27ac peaks (E) and differentially expressed genes (F) between normal and FLHS chickens, respectively. Significant threshold of differential peaks or genes was determined with |log2(FoldChange)|≥1 and p<0.05). FLHS, fatty liver hemorrhage syndrome.

Next, to elucidate the epigenetic and transcriptional changes induced by HFD in FLHS chickens, we first conducted data analysis of H3K27ac ChIP-Seq and found that the FLHS group showed a decrease in genome-wild H3K27 acetylation at 1 kb near transcription start site (TSS) and transcription termination site (TES) (Figure 3A–3B). Meanwhile, we also performed differential H3K27ac peaks and DEGs identification between control (CTR) and FLHS groups through DeSeq2. And the results show that 5.5% of all peaks were characterized by FLHS-related differential H3K27ac peaks, including 1.8% hyper-acetylated and 3.7% hypo-acetylated FLHS-related peaks (threshold: |log2(FoldChange)|≥ 1 and p≤0.05). And 4.0% of the differentially expressed genes (DEGs) of which 1.7% and 2.6% were up- and down-regulated in FLHS chickens (threshold: |log2(FoldChange)|≥1 and p≤0.05) (Figure 3C–3D; Supplementary Table S2). Among those, the Top 50 significant differential peaks and genes were shown in Figure 3E–3F, respectively. These results revealed HFD-induced FLHS chickens showed a large number of alterations in epigenomics and transcriptomics.

### Integrated analysis of ChIP-Seq and RNA-Seq

**Figure 4:**
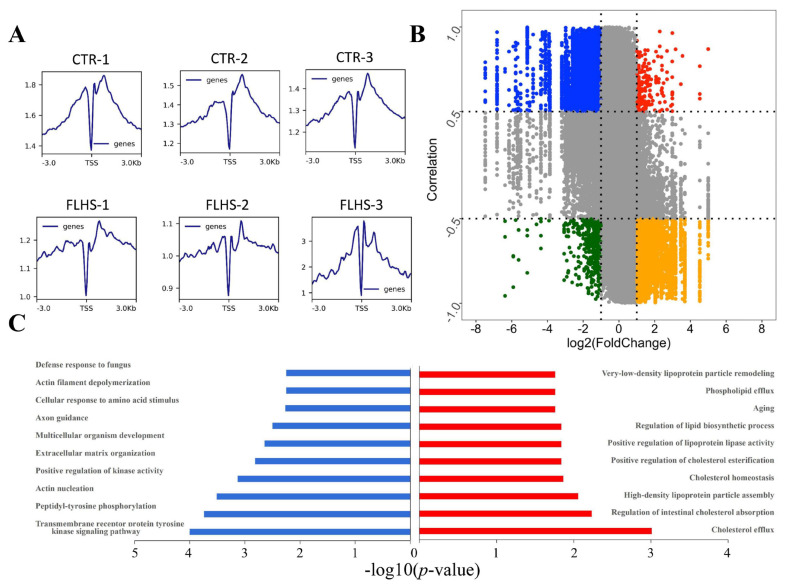
Integrated analysis of ChIP-Seq and RNA-Seq. (A) Profiles of H3K27ac histone modification peaks binding to the TSS from −3 kb to 3 kb (n = 3/group). (B) Genome-wide “four-way” plot showed the genes with a |Cor (peak-gene correlation) | ≥0.5 and a |log2(FoldChange)| ≥1 generated by integrated analysis of ChIP-Seq and RNA-Seq between CTR and FLHS groups. PP peak-genes are colored red, PN peak-genes are colored blue, NP peak-genes are colored yellow, and NN peak-genes are colored green. (C) Biological processes of putative target-genes of differentially hypo-acetylated peaks (left columns) and hyper-acetylated peaks (right columns) by DAVID (https://david-d.ncifcrf.gov/). CTR, control; FLHS, fatty liver hemorrhage syndrome.

Considering H3K27ac modification was enriched in the vicinity of the TSS in both normal and FLHS individuals to enhance gene expression as previous research [8] (Figure 4A). We performed an integrated analysis of ChIP-Seq and RNA-Seq to further investigate epigenetic changes linked to target genes. We adopted genome-wide “four way” to identify the peak-gene sets of PP and PN, which included 56 and 199 peak-genes in PP and PN, respectively as demonstrated in Figure 4B. Then, DAVID was adopted to further investigate the GO terms and pathways associated with differentially acetylated PP and PN peak-genes to understand the functional change induced by HFD (Figure 4C). Intriguingly, we found that the PN peak-genes were mainly involved in the peptidyl-tyrosine phosphorylation, biological processes of transmembrane receptor protein tyrosine kinase signaling pathway, actin nucleation etc., while the PP peak-genes were contributing to lipid metabolism such as cholesterol efflux, Cholesterol homeostasis, regulation of intestinal cholesterol absorption and high-density lipoprotein particle assembly etc. Moreover, MAPK and PPAR signaling pathways were the most significant pathways enriched in PN and PP peak-genes, respectively shown in Table 1. Table 2 exhibits the representatively up- and down-regulated peak-genes enriched in the biological processes related to lipid metabolism, apoptosis and inflammation, including *PCK1* (Chr20:12253024-12255050) [22], *APOA1* (Chr24:6035192-6037087) [23], *APOA4* (Chr24:6035192-6037087) [24], *NCP2* (Chr5: 38130423-3813087) [25], *FABP1* (Chr4:860 39840-86044093) [26]), and immune processes of *NTRK2* (ChrZ:41070698-41075835) [27], *KIT* (Chr4:65768031-65771957) [28], *PDGFRA* (Chr4:64724065-64728188) [29], *EPHA4* (Chr9:8608108-8609299) [30], and *ROR1* (Chr8: 29046548-29051340) [31]), respectively.

### Potential super-enhancers associated with FLHS

**Figure 5:**
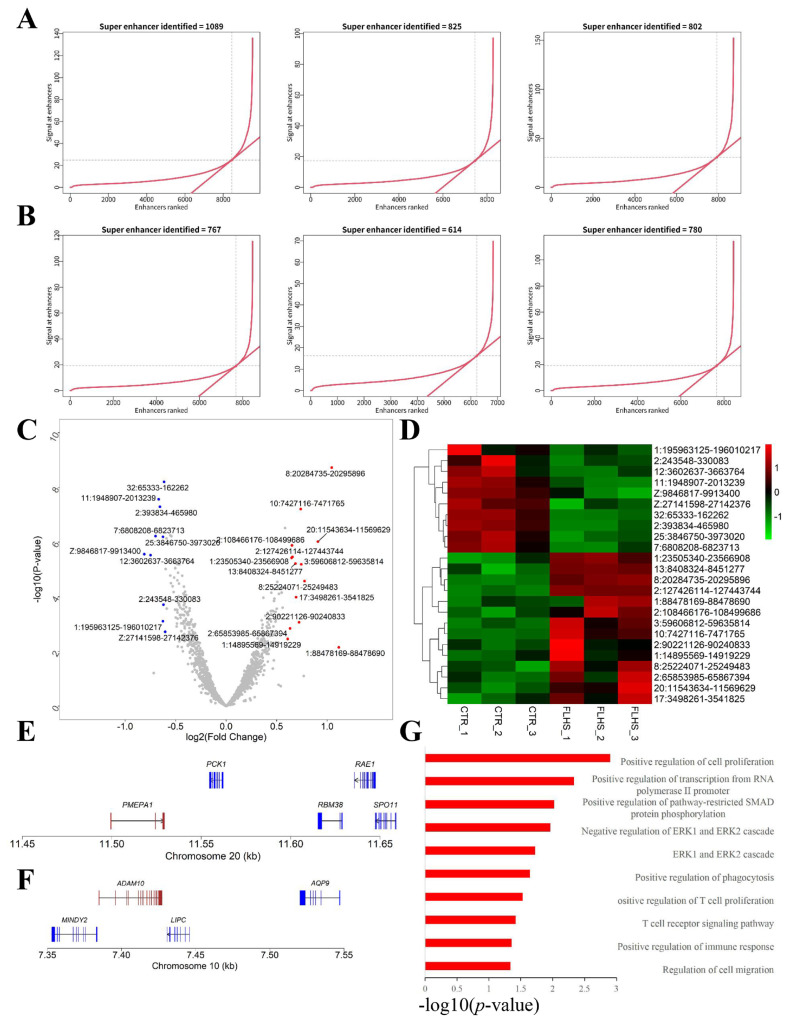
Analysis of super-enhancers between CTR and FLHS groups (A–B) The distribution of super-enhancers (SEs) was analyzed in control individuals (A) and individuals with FLHS (B). Enhancers above the inflection point of the curve were classified as SEs. (C) Volcano plot illustrated the differential SEs between the control and FLHS groups. The differential SEs were determined in |log2(FoldChange)| ≥0.6 and p<0.05. Dots in the right represented up-regulated SE peaks, left dots indicated down-regulated SE peaks, and dots in the center signified no significant difference. (D) Heatmaps displayed the significantly differential SEs between the CTR and FLHS groups (n = 3/group). (E–F) The activity track of H3K27ac in two representative SE peaks. (G) Enrichment analysis of up-regulated SE-associated genes (https://david-d.ncifcrf.gov/). CTR, control; FLHS, fatty liver hemorrhage syndrome.

As previously described that the ability of a super enhancer to specifically drive a single biological outcome which is critical to cell type-specific function, and the specific biological process was found to be associated with liver diseases such as alcoholic hepatitis and hepatocellular carcinoma [6,11]. Therefore, we further performed an analysis of super enhancer to explore the difference analysis of SEs in CTR and FLHS groups. A total of 1,089, 825, and 802 SEs in CTR individuals were revealed, respectively; while a total of 767, 614, and 780 SEs in FLHS individuals were identified, respectively (Figure 5A–5B). Then we merged the SE peaks presented in CTR and FLHS groups with bedtools (merge-i), respectively; for further adopting DEseq2 to analyze the differential SEs in FLHS group compared to CTR group. As shown in Figure 5C, we identified 10 down-regulated SEs and 14 up-regulated SEs in CTR and FLHS groups, respectively. (threshold: |log2(FoldChange)|≥ 0.6 and p-value≤ 0.05). The significantly up- and down-regulated SE peaks are presented in Figure 5D. Furthermore, we obtained the normalized signal of locations and found that differential SEs cover or are adjacent to genes of lipid metabolism process, which were used for further validating the differential activity of SEs between CTR and FLHS groups. For instance, the SE (Chr 10:7427116-7471765) with higher enrichment in FLHS group was identified in close proximity to *PCK1* which is mentioned in Table 2, and the SE (Chr20:11543634-11569629) with increased hyper-acetylation in FLHS groups near *AQP9* was reported to associate with lipid metabolism [32] (Figure 5E–5F). Interestingly, the functional enrichment of nearby genes annotated by up-regulated SEs was associated with immune response including positive regulation of immune response, positive regulation of T cell proliferation and positive regulation of phagocytosis presented in Figure 5G. Together, these results sufficiently demonstrated that SEs play an important role in regulating disease-associated genes and biological function.

### Comparative analysis of fatty liver disease in three different species

**Figure 6:**
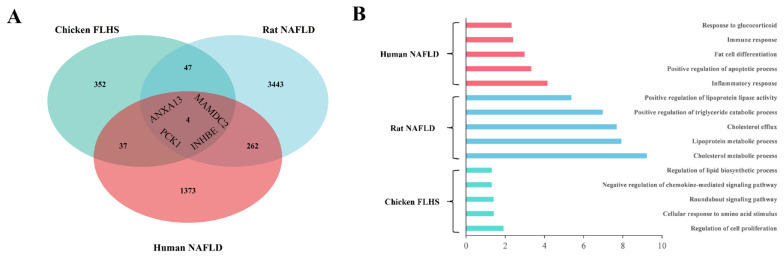
Comparative analysis of fatty liver disease in human, rat, and chicken in RNA expression level. (A) Venn diagram showing overlap of DEGs among Human, Rat and chicken. (B) Enrichment of biological processes of DEGs among three differential species by DAVID (https://david-d.ncifcrf.gov/). (DEGs, p≤0.05 and |log2FoldChange|≥1). DEGs, differentially expressed genes.

As previous research reported the close relationship between NAFLD and FLHS [2], we compared the similarities and differences between FLHS and NAFLD by downloading RNA-Seq data from human (GSE135251) and rat GSA (https://ngdc.cncb.ac.cn/gsa/CRA002638), combined with our RNA-seq data from chicken. Then we identified 3,756 DEGs in rat and 1,676 DEGs in human by adopting DeSeq2 R package (DEGs, |log2(FoldChange)| ≥1 and p-value≤0.05). Interestingly, four conservative genes (*MAMDC2*, *ANXA13*, *PCK1*, *INHBE*) intersected in these three species (Figure 6A), among which the up-regulated genes of *PCK1* [22] and *INHBE* [33] could promote lipid accumulation while the metabolism-related function of down-regulated genes of *ANXA13* and *MAMDC2* remained obscure. Subsequently, functional enrichment analysis was performed with DAVID, and the representative biological processes including “Response to glucocorticoid (GO:0051384)”, and “Fat cell differentiation (GO:0045444)” were significantly enriched in human, while “Positive regulation of lipoprotein lipase activity (GO:0051 006)”, “Positive regulation of triglyceride catabolic process (GO:0010898)”, “Cholesterol efflux (GO:0033344)”, “Lipoprotein metabolic process (GO:0042157)”, “Cholesterol metabolic process (GO:0008203)” were overrepresented in rat, and “Regulation of lipid biosynthetic process (GO:004 6890)” were present in chicken (Figure 6B).

### Validation of potential hub genes

To confirm the potential hub genes highly associated with FLHS, we first sought out the peak-associated target genes through functional enrichment analysis of peak-genes. We revealed that PPAR signaling pathway (close related to lipid metabolism) [34,35] and MAPK signaling pathway (close related to apoptosis, cell proliferation, senescence and differentiation) [36] were the most significant pathways in up- and down-regulated peak-genes, respectively (Table 1). Interestingly, the members of PPAR signaling pathway, *APOA1* and *PCK1* were often enriched in biological processes involved in lipid metabolism together with *APOA4*, and were related to lipid metabolic disorders [23,24,37]. Besides, *NTRK2* and *KIT* are members of the MAPK signaling pathway associated with cell apoptosis [27,28].

Subsequently, a comparative analysis of fatty liver disease was conducted and four conservative genes including *MAMDC2*, *ANXA13*, *PCK1*, and *INHBE* were identified intersection in DEGs of human, rat, and chicken (Figure 6A), indicating that these genes may play an important role in fatty liver disease no matter which species. Therefore, we suggested that the genes mentioned above, including five peak-related genes (*PCK1*, *APOA4*, *APOA1*, *NTRK2*, and *KIT*) and four conservative genes (*PCK1*, *INHBE*, *MAMDC2*, and *ANXA13*), are potential hub genes for the pathogenesis and treatment of FLHS (Table 3). Among that, *PCK1* is of great importance for its peak-related hub genes, up-regulated SE coverage genes, and conserved genes.

**Figure 7:**
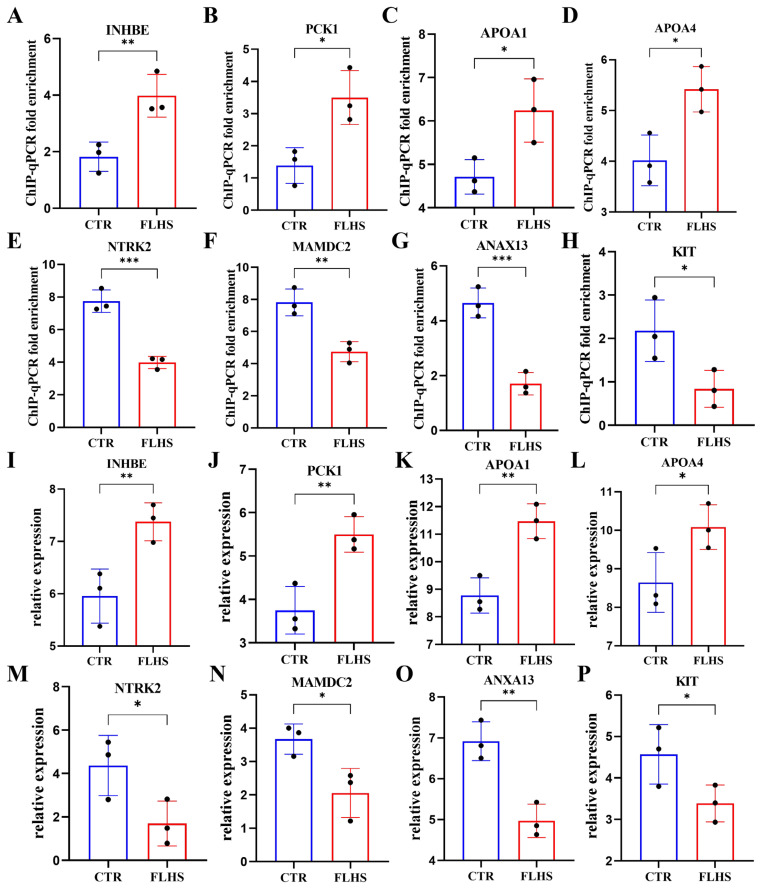
ChIP-qPCR and RT-PCR analyses in potential hub genes. (A–H) ChIP-qPCR fold enrichment of *INHBE*, *PCK1*, *APOA4*, *APOA1*, *NTRK2*, *MAMDC2*, *ANXA13* and *KIT* genes in FLHS chickens compared to normal individuals. (I–P) The relative mRNA expression levels of *INHBE*, *PCK1*, *APOA4*, *APOA1*, *NTRK2*, *MAMDC2*, *ANXA13* and *KIT* genes. The significance was assessed using a t-test. * p≤0.05, ** p≤0.01, and *** p≤0.001. RT-PCR, real-time polymerase chain reaction; CTR, control; FLHS, fatty liver hemorrhage syndrome.

To further validate these hub genes, we used RT-PCR and ChIP-qPCR to detect the expression and H3K27ac enrichment levels of *PCK1*, *APOA1*, *INHBE*, *APOA4*, *KIT NTRK2*, *MAMDC2*, *ANXA13* genes in normal and FLHS individuals. A significant increase (p-value≤ 0.05) in ChIP-qPCR fold enrichment of *PCK1*, *APOA1*, *INHBE*, and *APOA4* was observed in FLHS chickens, while *KIT NTRK2*, *MAMDC2*, and *ANXA13* were significantly decreased (p-value≤0.05) (Figure 7A–7H). The results of RT-PCR remained consistent, further proving the validity of our hypothesis (Figure 7I–7P).

## DISCUSSION

In this study, we initially identified the interaction of FLHS in transcriptome and histone acetylome alterations. Firstly, we found that HFD-induced FLHS chickens exhibited many differential acetylated peaks and differentially expressed genes compared to normal chickens. Then we constructed a Genome-wide “four-way” analysis to integrate ChIP-seq and RNA-seq to obtain peak-associated genes. Intriguingly, we identified peak-genes of up-regulation (*PCK1* [22], *APOA4* [24], *APOA1* [23], *NCP2* [25], *FABP1* [26]) and down-regulation (*ROR1* [31], *EPHA4* [30], *PDGFRA* [29], *KIT* [28], *NTRK2* [27]), which were significantly enriched in the biological processes of lipid metabolism, apoptosis, and inflammation. Moreover, functional enrichment analysis of PN (upregulated peak-genes positively regulated by H3K27ac) and PP (downregulated peak-genes positively regulated by H3K27ac) genes showed that the most significant pathways were MAPK and PPAR signaling pathways, respectively. As previously reported, PPAR signaling pathway is divided into PPARα, PPARβ/δ and PPARγ. Among those, PPARα could control the gene expression level of peroxisomal β-oxidation rate limiting enzymes to regulate hepatic lipid and plasma lipoprotein metabolism to avoid excessive lipid accumulation and prevent the progression of NAFLD [34]. While PPARγ could promote lipid droplet formation and hepatic lipid uptake, further promoting the damage of hepatocytes [35]. Moreover, it is known that the MAPK signaling pathway contributes to cytokine induced cell apoptosis [36]. And there could be potential interactions or crosstalk between MAPK and PPAR signaling pathways. For instance, MEK1/MAPK can downregulate PPARγ by reducing its ability to transactivate nuclear target genes, thereby inhibiting its genomic function [38]. Additionally, previous reports have indicated that both MAPK and PPAR signaling pathways play roles in inflammation. The knockdown of SEMA7A was shown to inhibit apoptosis and inflammation by activating PPAR-γ and inactivating MAPK in Parkinson’s disease [39]. Furthermore, Phellinus linteus polysaccharide demonstrated anti-inflammatory functions, and its molecular mechanism involves MAPK and PPAR signal pathways, leading to the reduction of inflammatory cytokine expressions [40]. Together, these findings indicate that HFD triggered histone modification of H3K27ac, subsequently altering gene expression. The genes affected are primarily involved in mechanisms of excessive lipid deposition and the dysregulation of apoptosis, leading to the imbalance of MAPK and PPAR signaling pathways. Moreover, the dysregulated MAPK and PPAR signaling pathways may interact and further contribute to the pathogenesis of FLHS through inflammation and apoptosis.

Super enhancer has been reported to be involved in regulating the specific biological process of liver diseases [6,11], while there were few studies in FLHS. To broaden our view of the influence of super enhancer on FLHS, we used H3K27ac ChIP-seq data to analyze super enhancer with different activities. Interestingly, two significant up-regulated SEs coverage genes, including *PCK1* and *AQP9* are involved in lipid metabolism. Among that, *PCK1*-mediated phosphorylation of *INSIG1/2* could promote the activation of *SREBP1* lipogenesis [37], and *AQP9* could facilitate the hepatic uptake of glycerol and knockdown of the *AQP9*, thereby reducing hepatic steatosis [32]. These results suggested that H3K27 acetylation alterations not only at the level of the active enhancer but also at the super enhancers level in HFD-induced FLHS model, which implied the importance of super enhancer in the pathogenesis of FLHS; and these genes covered in significantly dysregulated SEs may be potential targets for FLHS.

In addition, to further elucidate the key genes in the progression of fatty liver disease in different species, we conducted the comparative analysis of RNA-seq data to identify conservative genes in human, rat, and chicken. Interestingly, we found four DEGs related to lipids, including *INHBE* [33], *PCK1* [22], *ANXA13*, *MAMDC2* which overlapped in RNA-seq data of human NAFLD, rat NAFLD and chicken FLHS. Functional enrichment analysis revealed that Human DEGs are mainly enriched in processes of fat cell differentiation, positive regulation of apoptotic process, inflammatory response, and immune response, while Rat and Chicken DEGs tend to overrepresent in the processes of lipoprotein/cholesterol metabolic, cholesterol efflux and lipid biosynthetic process, suggesting the vital role of lipid metabolic process in different species of fatty liver disease. Altogether, these four conservative genes and lipid metabolism may be a key contributor to fatty liver disease in different species.

Herein, we further concluded potential hub genes including five peak-associated genes of *PCK1*, *APOA4*, *APOA1*, *KIT*, *NTRK2* and four conservative genes of *PCK1*, *INHBE*, *MAMDC2*, *ANXA13* (Table 3), which were critical for FLHS and may become potential therapeutic targets. Intriguingly, *PCK1* overlapped in integrative analysis of peak-associated genes, analysis of SE target genes and comparative analysis of conservative genes. *PCK1* is the first rate-limiting enzyme for hepatic glucose isomerization and mediates glycerol isomerization [41]. The latest study found that liver *PCK1* deficiency exacerbates lipid deposition in male mice with NAFLD. Interestingly, systemic knockdown of *PCK1* prevents liver inflammation [42]. *APOA4* was known as promoting lipid accumulation, and overexpression of *APOA4* may lead to increase the secretion of cholesteryl ester, phospholipids, and TG in chylomicron particles [24]. *INHBE* associates body mass index with insulin resistance, and knockdown *INHBE* with siRNA could inhibit body weight gain by diminished fat [33,43]. While *APOA1* possessed an anti-obesity effect which is associated with the increase of energy expenditure [23]. Thus, up-regulated genes including *PCK1*, *APOA4*, *INHBE* associated with lipid accumulation may be induced by HFD to promote lipid deposition, while up-regulated genes of *APOA1* may be the body’s compensation against the impact of HFD. Among downregulated genes (*KIT*, N*TRK2*, *MAMDC2*, and *ANXA13*), *KIT* could regulate apoptosis [28], *NTRK2* was revealed that capable of activating apoptosis pathways with Brain derived neurotrophic factor (*BDNF*) [27], which may promote the progression of fatty liver disease. However, the functions of *MAMDC2* and *ANXA13* in lipid metabolism disorders are still unclear.

**Figure 8:**
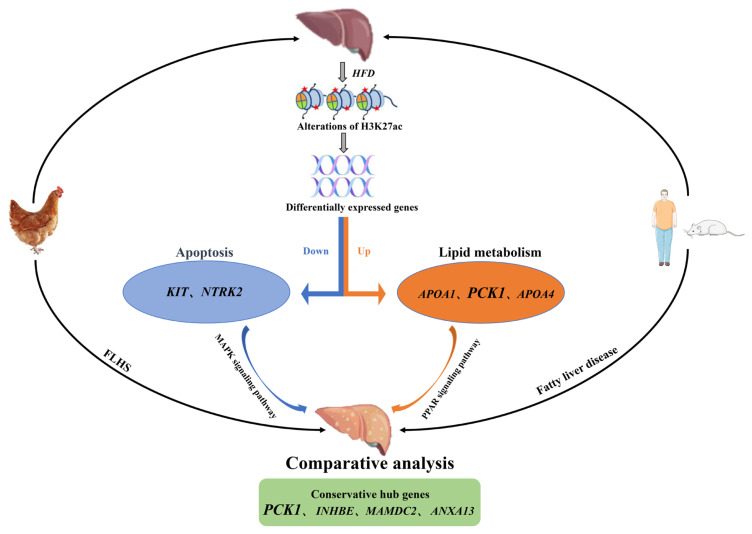
A brief proposal model of altered epigenetic regulation mechanisms of histone H3K27 acetylation causing FLHS and a summarized workflow of comparative analysis of fatty liver disease among different species. HFD affected histone modification of H3K27ac and resulted in dysregulation of genes evolving in lipid metabolism (*PCK1*, *APOA4*, *APOA1*) and apoptosis (*KIT*, *NTRK2*) together with PPAR signaling pathway and MAPK signaling pathway, which may induce the progression of FLHS. Meanwhile comparative analysis of human and rat with fatty liver together with FLHS chicken revealed four conservative hub genes (*PCK1*, *INHBE*, *MAMDC2*, *ANXA13*), especially for *PCK1*, which may play a vital role in fatty liver disease. FLHS, fatty liver hemorrhage syndrome; HFD, high-fat diet.

Subsequently, based on the functions of candidate genes and previous studies on MAPK and PPAR enrichment pathways, an epigenetic mechanism model for FLHS chickens was proposed, and comparative analysis was conducted on different species of fatty liver disease (Figure 8). In this model, the HFD may trigger histone modification of H3K27ac, leading to dysregulation of candidate genes associated with lipid metabolism (*PCK1*, *APOA4*, *APOA1*) and apoptosis (*KIT*, *NTRK2*) together with PPAR and MAPK signaling pathways, which results in lipid metabolic disorders and dysregulation of apoptosis inducing the formation of FLHS. Moreover, four conservative genes (*PCK1*, *INHBE*, *MAMDC2*, *ANXA13*) intersected in species of human, rat, and chicken, especially for *MAMDC2* and *ANXA13*, which were under further experimental investigation. The whole epigenetic regulatory work of this study enhances our understanding of epigenetic mechanisms of FLHS, and processes of comparative analysis provide insights on fatty liver disease, which further develop pathogenesis and potential therapeutic strategy and biomarkers for fatty liver disease.

To the best of our knowledge, our study is the first to provide genome-wide association analysis at the epigenetic levels and transcriptional levels, as well as comparative levels for screening the conservative pivotal genes to further enhances our understanding of the etiology and mechanism of FLHS. However, it is quite important to propose our limitations in our current study. On the one hand, there are only limited sequenced samples, which is possibly leading to a degree of fortuity in our research; on the other hand, further experiments about the key marker genes and molecular biological processes and pathways are needed to be confirmed.

Our study elaborated extensively on H3K27ac alterations, exhibiting active enhancers and SEs that lead to HFD-induced FLHS chickens, and performed a comparative analysis of fatty liver disease in multi species. It is worth noting that we have identified hub genes associated with lipid metabolism (*INHBE*, *PCK1*, *APOA4*, *APOA1*) and apoptosis (*KIT*, *NTRK2*) together with PPAR and MAPK signaling pathway, especially *PCK1*, which may be the pathogenesis and potential therapeutic targets of FLHS.

## Figures and Tables

**Table 1:** The most significant pathway in upregulated and downregulated peak-genes, respectively

ID	Pathways	p-value	Genes	Regulated	Function
gga03320	PPAR signaling pathway	1.87E-02	*FABP1*, *APOA1*, *PCK1*	UP	PPARα plays a role in the clearance of circulating or cellular lipids via the regulation of gene expression involved in lipid metabolism in liver. PPARγ promotes hepatic lipid uptake and lipid deposition
gga04010	MAPK signaling pathway	9.73E-04	*PDGFRA*, *NTRK2*, *FGF7*, *IL1R1*, *FGF19*, *PDGFD*, *NTF3*, *KIT*, *EREG*	Down	The MAPK cascade is a highly conserved module that is involved in various cellular functions, including apoptosis, cell proliferation, differentiation, and migration

PPAR, peroxisome proliferator-activated receptor; MAPK, mitogen-activated protein kinase.

**Table 2:** Interested genes enriched in the biological process are related to lipid metabolism, apoptosis and inflammation

Gene	log2(FoldChange)	p-value	H3K27ac peaks	Correlation	Regulated	Function
*PCK1*	2.983	4.89E-02	Chr20:12253024-12255050	0.541	Up	This gene is the main control point for the regulation of gluconeogenesis, promotes lipogenesis
*APOA4*	1.839	5.24E-05	Chr24:6035192-6037087	0.543	Up	The protein encoded by this gene which constitutes chylomicron and transfers to HDL with TG lipolysis in chylomicron is associated with atherosclerosis, lipoprotein oxidation, glucose metabolism
*APOA1*	1.630	2.90E-02	Chr24:6035192-6037087	0.558	Up	The encoded precursor protein promotes cholesterol efflux from tissues to the liver for excretion and is a cofactor of lecithin cholesterol acyltransferase
*NCP2*	1.139	1.98E-05	Chr5: 38130423-38130871	0.577	Up	The protein encoded by this gene collaboratively works with NPC1 to regulate the egress of endocytosed cholesterol from late endosome/lysosome
*FABP1*	1.030	1.17E-03	Chr4:86039840-86044093	0.725	Up	This gene encodes a kind of liver-specific protein which plays an important role in lipid metabolism. And overexpression of FABP1 in the liver can inhibit lipid autophagy
*ROR1*	1.087	4.66E-03	Chr8:29046548-29051340	0.652	Down	This gene encodes a receptor tyrosine kinase (RTK) which is Wnt5a/b and Wnt16 receptor in Wnt signaling molecules and as a therapeutic target in a few tumors
*EPHA4*	1.145	1.18E-03	Chr9:8608108-8609299	0.801	Down	This gene encodes a receptor tyrosine kinase (RTK) which is related to inflammation.
*PDGFRA*	1.152	9.39E-04	Chr4:64724065-64728188	0.625	Down	It encodes a receptor tyrosine kinase responding to platelet-derived growth factor (PDGF) which is mutated associated with gastrointestinal stromal tumors (GISTs), inflammatory fibroid polyps, and gliomas
*KIT*	1.308	3.73E-02	Chr4:65768031-65771957	0.550	Down	This gene encodes a receptor tyrosine kinase (RTK) which is a part of signaling pathways for controlling multiple cellular processes including cell proliferation and apoptosis
*NTRK2*	2.850	1.15E-02	ChrZ:41070698-41075835	0.536	Down	It encodes a neurotrophic receptor tyrosine kinase 2 regulating cell apoptosis

*PCK1*, phosphoenolpyruvate carboxykinase 1; *APOA4*, apolipoprotein A4; *APOA1*, apolipoprotein A1; *NCP2*, NPC intracellular cholesterol transporter 2; *FABP1*, fatty acid binding protein 1; *ROR1*, receptor tyrosine kinase like orphan receptor 1; *EPHA4*, EPH receptor A4; *PDGFRA*, platelet derived growth factor receptor alpha; *KIT*, tyrosine-protein kinase kit; *NTRK2*, neurotrophic receptor tyrosine kinase 2.

**Table 3:** Potential target hub genes involving lipid metabolism and apoptosis etc

Gene symbol	H3K27ac	Log2(FoldChange)	p-value	Correlation	Function
Interested peak-associated target genes201-408-2558					
*APOA1*	24:6035192-6037087	1.630	2.90E-02	0.558	The encoded precursor protein promotes cholesterol efflux from tissues to the liver for excretion and is a cofactor of lecithin cholesterol acyltransferase
*APOA4*	24:6035192-6037087	1.839	5.24E-05	0.543	The protein encoded by this gene which constitutes chylomicron and transfers to HDL with TG lipolysis in chylomicron is associated with atherosclerosis, lipoprotein oxidation, glucose metabolism
*PCK1*	20:12253024-12255050	2.983	4.89E-02	0.541	This gene which could promote lipogenesis and caspase-dependent apoptosis is the main control point for the regulation of gluconeogenesis
*KIT*	4:65768031-65771957	−1.308	3.73E-02	0.550	This gene encodes a receptor tyrosine kinase (RTK) which is a part of signaling pathways for controlling multiple cellular processes including cell proliferation and apoptosis
*NTRK2*	Z:41070698-41075835	−2.850	1.15E-02	0.536	It encodes a neurotrophic receptor tyrosine kinase 2 regulating cell apoptosis
Genes intersected among different species of fatty liver disease					
*PCK1*	-	2.983	4.89E-02	-	This gene which could promote lipogenesis and caspase-dependent apoptosis is the main control point for the regulation of gluconeogenesis
*INHBE*	-	1.261	8.84E-04	-	A member of the TGF-beta (transforming growth factor-beta) superfamily of proteins encoded by this gene improves metabolic status under obese insulin-resistant and siINHBE suppresses body weight gain by diminishing fat
*ANXA13*	-	4.80E-03	−1.251	-	-
*MAMDC2*	-	4.31E-02	−2.226	-	-

H3K27ac, Histone H3 lysine 27 acetylation; *APOA1*, apolipoprotein A1; *APOA4*, apolipoprotein A4; *PCK1*, phosphoenolpyruvate carboxykinase 1; *KIT*, tyrosine-protein kinase kit; *NTRK2*, neurotrophic receptor tyrosine kinase 2; *INHBE*, inhibin subunit beta E; *ANXA13*, annexin A13; *MAMDC2*, MAM domain containing.

## Data Availability

All the sequencing data of RNA-Seq and ChIP-Seq will be deposited at GSA (https://ngdc.cncb.ac.cn/gsa/) upon acceptance. RNA-Seq data of fatty liver disease of human and rat from GEO (GSE135251) and GSA (https://ngdc.cncb.ac.cn/gsa/CRA002638), respectively.

## SUPPLEMENTARY MATERIAL

**Supplementary Table S1**. Summaries of ChIP-Seqand RNA-Seqexperiments

**Supplementary Table S2**. Percentageof differential acetylatedpeaks and dysregulated genes, respectively
