# Supplementary material for: Epigenetic regulation of key gene of PCK1 by enhancer and super-enhancer in the pathogenesis of fatty liver hemorrhagic syndrome
Source: Anim Biosci. 2024 Apr 23;37(8):1317–32. doi: 10.5713/ab.23.0423 (PMC11222861; doi:10.5713/ab.23.0423)
Supplement: Supplementary file 1 [file ab-23-0423-Supplementary-Table-1.pdf]

TABLE S1 | Summaries of ChIP-Seq and RNA-Seq experiments

ChIP-Seq

Sample	Input total mapped reads	Input unique mapped reads	Ac total reads	Ac unique mapped reads	Input unique mapped ratio	Ac unique mapped ratio	Peak number	Average peak length
CTR_1	38747204	34940031	35192454	31918148	90.2%	90.7%	25742	4660
CTR_2	39174785	35539369	35310289	32254513	90.7%	91.3%	27556	4151
CTR_3	39701029	36383673	42619654	39138069	91.6%	91.8%	26982	4246
FLHS_1	32699236	29543522	35007261	31895273	90.3%	91.1%	23519	3368
FLHS_2	34065472	30950795	32945926	29712239	90.9%	90.2%	22110	3154
FLHS_3	31610272	28974731	32826167	30120421	91.7%	91.8%	21645	2812
Mean	35999666	32722020	35650292	32506444	90.9%	91.2%	24592	3732

RNA-Seq

Sample	Total mapped reads	Unique mapped reads	Unique mapped ratio	Multiple mapped ratio
CTR_1	49152854	47559262	96.8%	1.0%
CTR_2	48533802	45681913	94.1%	1.2%
CTR_3	51647162	49514232	95.9%	1.1%
FLHS_1	51043166	48555943	95.1%	1.3%
FLHS_2	52883657	51094574	96.6%	1.2%
FLHS_3	49365533	46618056	94.4%	1.2%
Mean	49894158	47544758	95.3%	1.1%
