# Supplementary material for: Epigenetic regulation of key gene of PCK1 by enhancer and super-enhancer in the pathogenesis of fatty liver hemorrhagic syndrome
Source: Anim Biosci. 2024 Apr 23;37(8):1317–32. doi: 10.5713/ab.23.0423 (PMC11222861; doi:10.5713/ab.23.0423)
Supplement: Supplementary file 2 [file ab-23-0423-Supplementary-Table-2.pdf]

TABLE S2 | Percentage of differential acetylated peaks and dysregulated genes, respectively

ChIP-Seq (Total number =24179)		RNA-Seq (Total number =14753)	
hyper-acetylated peaks	hypo-acetylated peaks	up-regulated genes	down-regulated genes
1.8%	3.7%	1.7%	2.6%
